# Was low CO_2_ a driving force of C_4_ evolution: *Arabidopsis* responses to long-term low CO_2_ stress

**DOI:** 10.1093/jxb/eru193

**Published:** 2014-05-22

**Authors:** Yuanyuan Li, Jiajia Xu, Noor Ul Haq, Hui Zhang, Xin-Guang Zhu

**Affiliations:** ^1^State Key Laboratory of Hybrid Rice Research, Shanghai Institute of Biological Sciences, Chinese Academy of Sciences, Shanghai 200031, China; ^2^Key Laboratory of Computational Biology and Partner Institute for Computational Biology, Chinese Academy of Sciences, Shanghai 200031, China; ^3^Shandong Normal University, Jinan, Shandong 250014, China

**Keywords:** *Arabidopsis*, C4 photosynthesis, evolution, low CO2, photorespiration, stress responses.

## Abstract

The responses of long-term growth of plants under elevated CO_2_ have been studied extensively. Comparatively, the responses of plants to subambient CO_2_ concentrations have not been well studied. This study aims to investigate the responses of the model C_3_ plant, *Arabidopsis thaliana*, to low CO_2_ at the molecular level. Results showed that low CO_2_ dramatically decreased biomass productivity, together with delayed flowering and increased stomatal density. Furthermore, alteration of thylakoid stacking in both bundle sheath and mesophyll cells, upregulation of PEPC and PEPC-K together with altered expression of a number of regulators known involved in photosynthesis development were observed. These responses to low CO_2_ are discussed with regard to the fitness of C_3_ plants under low CO_2_. This work also briefly discusses the relevance of the data to C_4_ photosynthesis evolution.

## Introduction

The response of plants grown in lower CO_2_ concentrations has been much less studied than responses to elevated CO_2_ concentrations (Long *et al.*, 2004, 2006; Ainsworth and Long, 2005; Gerhart and Ward, 2010). Among these limited studies, some have demonstrated that a large genetic variation in response to low CO_2_ exists among *Arabidopsis* accessions. For example, Sharma *et al.* (1979) screened 33 *Arabidopsis* accessions for survival time under limiting CO_2_ when grown side by side with C_4_ plants (*Zea mays* L.) in an air-tight chamber where CO_2_ concentration was reduced to below the compensation point of C_3_ plants and found a 1–2-week difference in the survival time in different accessions and also found substantial genetic segregation among F_2_ parents, with extreme differences in survival time near the CO_2_ compensation point.

*Arabidopsis* genotypes from different elevations show significant variation in the response of seed number when grown at low CO_2_ (20 Pa) (Ward and Strain, 1997). Ward *et al.* (2000) performed an artificial selection experiment using *Arabidopsis* for high seed number over five generations at low CO_2_ (20 Pa, or 200 ppm); the selected populations produced 25% more seeds and 35% more biomass on average than control populations which were randomly selected at the fifth generation when grown at low CO_2_. In addition, Ward and Kelly (2004) also observed a high level of genetic variation in survival, reproductive output, and total seed production among the *Arabidopsis* genotypes when grown at low CO_2_ (200 ppm). All these studies suggest that *Arabidopsis* has adaptive phenotypic plasticity in response to low CO_2_.

In a carbon starvation experiment, 5-week-old *Arabidopsis* rosettes treated with ambient (350 ppm) CO_2_ or compensation point (<50 ppm) CO_2_ were collected in the light for 4h to investigate responses to changing endogenous sugar concentrations in rosettes at the gene expression level using the GeneChip *Arabidopsis* ATH1 genome array (Bläsing *et al.*, 2005). However, these studies have not addressed the mechanism of long-term responses of plants to low CO_2_.

This study conducted a survey of responses of C_3_ plants to long-term low CO_2_ treatments at the molecular level. *Arabidopsis* was chosen as the model system because its genome has been fully sequenced and is still the best annotated plant genome to date (The Arabidopsis Genome Initiative, 2000); the well-annotated *Arabidopsis* genome facilitates analysis of global gene expression using RNA-Seq technology. This study sequenced the transcriptome of 6-week old *Arabidopsis* seedlings grown under ambient CO_2_ (380 ppm) or low CO_2_ (100 ppm). The results are discussed with particular reference to the significance of the altered gene expression to the fitness of C_3_ plants under low CO_2_. The relevance of low CO_2_ to C_4_ evolution is also briefly discussed.

## Materials and methods

### Plant growth and harvest

*Arabidopsis thaliana* Columbia-0 (Col-0) seeds were imbibed in 0.1% (w/v) agar solution and incubated at 4 °C for 2 d to break dormancy. Imbibed seeds were germinated and grown in Pindstrup soil in a Percival incubator (NC-350HC-LC, Nihonika, Japan) in which CO_2_ gas can be accurately and stably controlled in the range of 100–3000 ppm. CO_2_ concentrations 100 and 380 ppm were applied in two separate chambers and maintained throughout this study. CO_2_ concentrations were monitored and maintained throughout the experiments. Plants were grown under a 8/16h light/dark cycle (photosynthetic photon flux density 150 μmol m^–2^ s^–1^) at 21 °C and 70% relative humidity. After 4 weeks, the photoperiod was changed to a 16/8h light/dark cycle for a further 2 weeks. On day 42, samples were taken during the middle of the light period and mature expanded rosette leaves from 10–15 individual plants were harvested, immediately frozen in liquid nitrogen, and stored at –80 °C until use. The samples were taken from 12 individual pots.

### Morphological data collection

Scanning electron microscopy and transmission electron microscopy were used to observe the changes of ultrastructure by low CO_2_. The number of stomata was counted in four fields of view from the fully expanded leaves of no less than eight individual plants for each treatment (Supplmentary Fig. S1 available at *JXB* online).

### RNA preparation and sequencing

Total RNA was prepared with TRIzol (Invitrogen Life Technologies, Shanghai, China), according to the manufacturer’s instructions. Following extraction, total RNA was purified using a RNeasy Mini Kit including on-column DNase digestion (Qiagen, Shanghai, China). Purified RNA was checked for integrity and quality using an Agilent 2100 Bioanalyzer (Agilent Technologies, Santa Clara, CA, USA). The cDNA library was constructed for sequencing as described in Illumina TruSeqTM RNA sample preparation version 2 guide (catalog no. RS-930–1021). Sequencing was performed using a Illumina HiSeq 2000 (Illumina, San Diego, USA).

### Mapping and quantification of sequence reads

Clean reads were mapped onto the latest *A. thaliana* Col-0 genome assembly (TAIR 10) or a minimal set of coding sequences of the TAIR 9 genome release (Gowik *et al.*, 2011) using the bowtie version 0.12.7 (Langmead *et al.*, 2009). The best hit of each read with a maximum of three nucleotide mismatches was used (-v 3 --best). The raw digital gene expression counts were normalized using the RPKM (reads/kb/million) method (Mortazavi *et al.*, 2008; Nagalakshmi *et al.*, 2008; Supplementary Tables S1 and S2 available at *JXB* online).

To identify differentially expressed genes, an expression profile matrix was built which integrated the digital gene expression count for each gene in each library, total gene count for each condition were used as background to check if a gene is significantly differentially expressed in low and CO_2_ normal conditions by applying the chi-squares test. A FDR-corrected *P*-value was calculated using the formula $q(i)=\frac{p(i)N}{i}C(N)$ where *i* represents the ascending order of *P*-values, *p*(*i*) represents the *i*th *P*-value, *C* represents a chosen constant, and *N* represents the size of dataset (Benjamini and Hochberg, 1995). Significantly differentially expressed genes were picked following the criteria *P*<0.001, FDR<0.025, |log_2_Ratio|≥1.2.

## Results

### Effects of long-term low CO_2_ on biomass growth, stomata density, and chloroplast ultrastructure

**Fig. 1.**
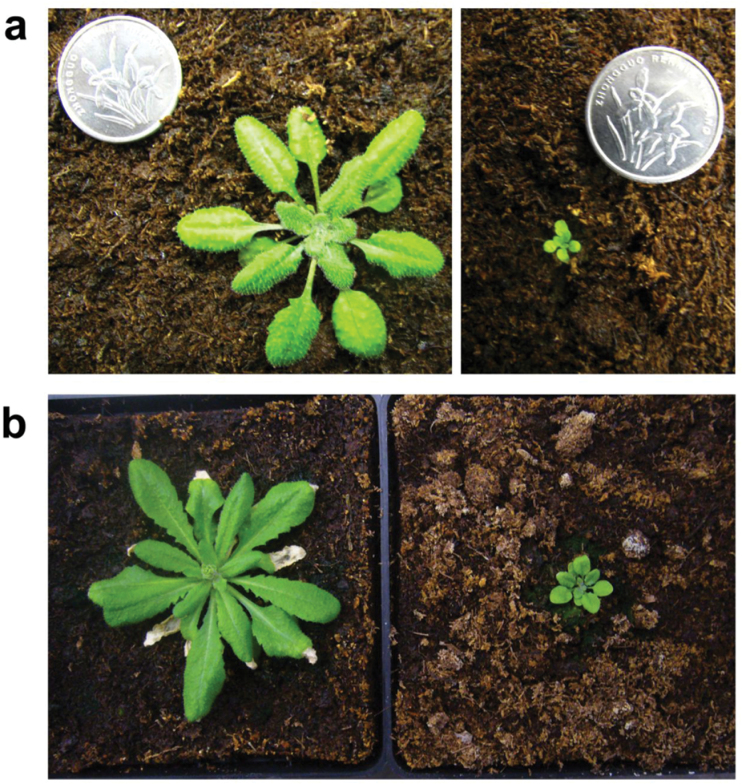
*Arabidopsis thaliana* Col-0 grown under normal CO_2_ (380 ppm) and low CO_2_ (100 ppm) for 4 weeks (A; 8/16h light/dark cycle (photosynthetic photon flux density 150 μmol m^–2^ s^–1^, 21 °C, 70% relative humidity) and for 6 weeks (B; 4 weeks under conditions as for A plus 2 weeks under a 16/8h light/dark cycle).

CO_2_ is the major source of carbon for photosynthesis and plays a vital role in plant growth. High CO_2_ often increases the growth and reproduction of C_3_ annuals, whereas low CO_2_ decreases growth (Ward *et al.*, 2000; Ward, 2005). Previous studies showed that minimum CO_2_ concentrations between 180 and 200 ppm during the Last Glacial Maximum were already stressful on modern C_3_ plants (Dippery *et al.*, 1995; Ward, 2005); therefore, this work set low CO_2_ concentration as 100 ppm. *Arabidopsis* plants grown at 100 ppm for 6 weeks were much smaller than those grown under normal CO_2_ (380 ppm) (Fig. 1). In addition, low CO_2_ led to a slight delay in flowering time (data not shown). The results showed that low CO_2_ (100 ppm) had a dramatic impact on the growth of the C_3_ plant *Arabidopsis*.

**Fig. 2.**
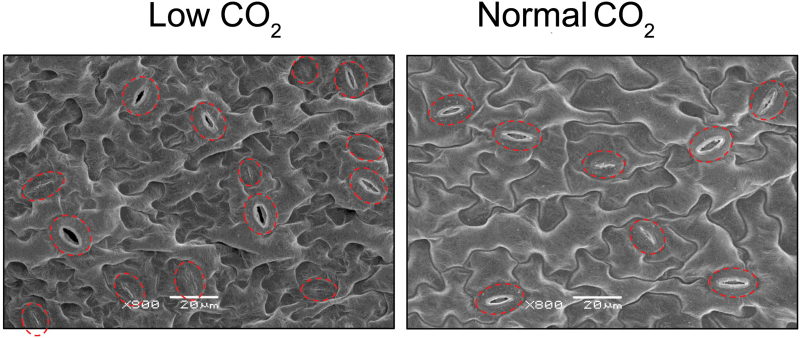
Effect of low atmospheric CO_2_ on stomatal density. Representative scanning electron micrographs of abaxial (lower) leaf blade epidermis of *Arabidopsis* grown under low CO_2_ (100 ppm) or normal CO_2_ (380 ppm) for 6 weeks. Dashed lines indicate stomata. Bars, 20 μm.

Stomata control the entry of CO_2_ into the leaves of plants for photosynthesis. There is a strong inverse correlation between atmospheric CO_2_ and stomatal density (the number of stomata per unit area) (Franks *et al.*, 2012). This work examined the stomatal density of abaxial (lower) leaf blade epidermis of *Arabidopsis* plants grown at either low CO_2_ or normal CO_2_ for 6 weeks (Supplmentary Fig. S1). As expected, stomatal density was significantly higher (mean±SE 509±59mm^–2^) in plants grown at low CO_2_ compared to plants at normal CO_2_ (297±54mm^–2^) (Fig. 2).

**Fig. 3.**
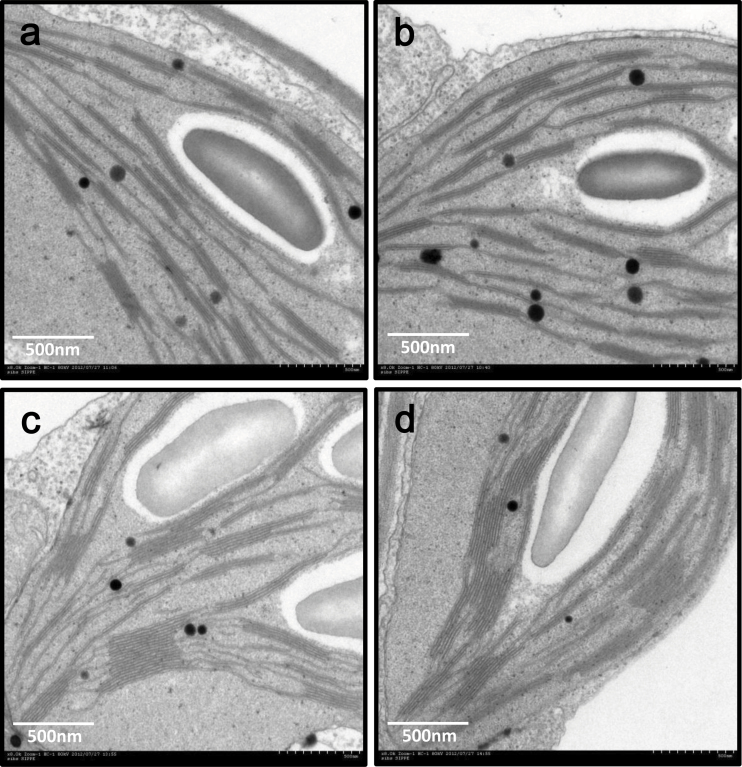
Effect of low CO_2_ on chloroplast ultrastructure. Representative transmission electron micrographs of ultrastructure of *Arabidopsis* grown under low CO_2_ (A, B) or normal CO_2_ (C, D) for 6 weeks: (A and C) mesophyll cell; (B and D) bundle sheath cell. Bars, 500nm.

In plants, photosynthesis occurs exclusively in the chloroplast, and the photosystems (PSI and PSII) exist on the thylakoid membrane inside a chloroplast. PSII is limited to granal thylakoids, while PSI exists exclusively in the thylakoids exposed to the stroma (Albertsson, 1995; Dekker and Boekema, 2005; Sakamoto *et al.*, 2008). The ultrastructure of mature leaves under low CO_2_ were examined using transmission electron microscopy, and the size and the arrangement of bundle sheath cells and mesophyll cells was not changed, while *Arabidopsis* grown under low CO_2_ showed decreased stacking in chloroplast grana in both mesophyll and bundle sheath cells under low CO_2_ compared to normal CO_2_ (Fig. 3).

### Some C_4_-cycle genes were upregulated under low CO_2_

The mRNA-seq analysis to compare transcriptomes between closely related C_4_ and C_3_ species within the genus *Flaveria* and *Cleome* using *Arabidopsis* as the reference genome defined a list of enzymes, transporters, and regulatory proteins required for the core C_4_ cycle (Bräutigam *et al.*, 2011; Gowik *et al.*, 2011). It has been reported that *Arabidopsis* shows the characteristics of C_4_ photosynthesis in midveins (Brown *et al.*, 2010), but nothing is known about the plasticity of these characteristics.

In order to check whether C_4_-related characteristics can be regulated by low CO_2_ stress, the transcript abundances of putative C_4_-related genes were examined. The transcript encoding the enzyme phosphoenolpyruvate carboxylase (PEPC, At2g42600) showed 2.10-fold higher transcript abundance, followed by PEPC kinase (PEPC-K, At1g08650) with a 1.99-fold increase in abundance (Table 1 and Supplementary Table S3 available at *JXB* online). In addition, the transcript abundances for the genes encoding alanine aminotransferase (At1g17290), chloroplast NAD-dependent malate dehydrogenase (At3g47520), pyruvate orthophosphate dikinase regulatory protein (At4g21210), inorganic pyrophosphatase 2 (At2g18230), chloroplast dicarboxylate transporter 1 (At5g12860) and 2 (At5g64280) showed trends of upregulation but their fold changes were less than 2.

**Table 1.** Transcription abundance of C_4_-cycle genes and C_4_-related transportersReads were mapped onto the latest *Arabidopsis thaliana* Col-0 genome assembly (gene mapping) or a minimal set of coding sequences of the TAIR 9 genome release (core set mapping) using bowtie. Low: low CO_2_, 100 ppm; Nor: normal CO_2_, 380 ppm; rpkm, reads per kilobase per million mapped reads. AlaAT, alanine aminotransferase; AspAT, aspartate amino transferase; cpNAD-MDH, chloroplast NAD-dependent malate dehydrogenase; Dit, chloroplast dicarboxylate transporter; PEPC, phosphoenolpyruvate carboxylase; PEPC-K, PEPC kinase; PEP-CK, PEP carboxykinase; PPA2, inorganic pyrophosphatase 2; PPDK-RP, pyruvate orthophosphate dikinase regulatory protein; PPT1, phosphoenolpyruvate/phosphate translocator 1; TPT, triose phosphate transporter; –, no expression detected.

Gene ID	Protein	Gene mapping			Core set mapping		
		Low (rpkm)	Nor (rpkm)	Fold change	Low (rpkm)	Nor (rpkm)	Fold change
At1g08650	PEPC-K	26.220	13.206	1.985	30.156	15.230	1.980
At1g17290	AlaAT	56.839	48.904	1.162	65.884	57.025	1.155
At1g62800	AspAT	0.707	2.388	0.296	0.916	2.701	0.339
At2g18230	PPA2	10.288	6.468	1.591	11.812	7.459	1.584
At2g42600	PEPC	197.604	93.979	2.103	227.099	108.523	2.093
At3g47520	cpNAD-MDH	74.044	64.394	1.150	85.015	74.261	1.145
At4g21210	PPDK-RP	213.075	191.911	1.110	244.822	221.204	1.107
At4g37870	PEP-CK	26.606	43.413	0.613	30.589	50.065	0.611
At5g12860	Dit1	356.959	275.153	1.297	409.991	317.354	1.292
At5g33320	PPT1	–	–	–	44.181	50.558	0.874
At5g46110	TPT	508.799	533.917	0.953	584.330	615.779	0.949
At5g64280	Dit2	27.003	20.059	1.346	–	–	–

### Photorespiratory genes showed trends of upregulation under low CO_2_

Low atmospheric CO_2_ concentration would increase photorespiration, so this work also examined the transcript abundances of photorespiration genes. Nearly all genes showed trends of upregulation in plants grown under low CO_2_ compared with those under normal CO_2_ (Table 2 and Supplementary Table S4 available at *JXB* online), except for the gene encoding glycine decarboxylase L-protein (*mtLPD1*; At1g48030); however, the fold changes were all less than 2. The differential responses of genes involved in the photosynthetic light reactions, Calvin Benson cycle, and ABA and IAA metabolisms were shown in Supplementary Tables S8–11 available at *JXB* online.

**Table 2.** Transcription abundance of photorespiration genesThe genes in bold represent these that plays a major function in photorespiration and the knockout results in a low CO_2_-sensitive phenotype (Bauwe, 2011). Reads were mapped onto the latest *Arabidopsis thaliana* Col-0 genome assembly (gene mapping) or a minimal set of coding sequences of the TAIR 9 genome release (core set mapping) using bowtie. Low: low CO_2_, 100 ppm; Nor: normal CO_2_, 380 ppm; rpkm, reads per kilobase per million mapped reads.

Gene ID	Enzyme	Gene	Gene mapping			Core set mapping		
			Low (rpkm)	Nor (rpkm)	Fold change	Low (rpkm)	Nor (rpkm)	Fold change
At1g11860	Glycine decarboxylase T-protein	*GLDT1*	909.564	789.090	1.153	1044.524	909.989	1.148
At1g23310	Glutamate:glyoxylate aminotransferase	*GGT1*	461.752	399.672	1.155	538.428	465.798	1.156
At1g48030	Glycine decarboxylase L-protein	*mtLPD1*	250.934	270.703	0.927	–	–	–
At1g68010	Hydroxypyruvate reductases	*HPR1*	302.977	286.059	1.059	347.873	329.958	1.054
At1g70580	Glutamate:glyoxylate aminotransferase	*GGT2*	39.407	23.536	1.674	–	–	–
At1g80380	l-Glycerate 3-kinase	*GLYK*	130.215	118.051	1.103	149.539	136.138	1.098
At2g13360	Alanine:glyoxylate aminotransferase	*AGT1*	1069.682	953.983	1.121	1228.446	1100.193	1.117
At2g26080	Glycine decarboxylase P-protein	*GLDP2*	87.272	75.611	1.154	216.751	173.881	1.247
At3g14415	Glycolate oxidase	*GOX2*	497.994	473.499	1.052	571.960	546.046	1.047
At3g14420	Glycolate oxidase	*GOX1*	698.380	617.984	1.130	801.954	712.742	1.125
At4g33010	Glycine decarboxylase P-protein	*GLDP1*	800.116	599.088	1.336	–	–	–
At4g37930	Serine hydroxymethyltransferase	*SHM1*	1188.138	842.873	1.410	1364.293	972.136	1.403

### Chloroplast biogenesis- and maintenance-related genes showed differential expression in low CO_2_

Given the differential expression of genes involved in chloroplast biogenesis and maintenance between the C_3_ and C_4_ *Flaveria* species (Gowik *et al.*, 2011) and the altered chloroplast ultrastructure between low CO_2_ and normal CO_2_ (Fig. 3), this work examined the transcript abundances of genes involved in chloroplast biogenesis and maintenance under low CO_2_ and compared them with previously identified genes differentially expressed between C_3_ and C_4_ species (Gowik *et al.*, 2011) (Table 3 and Supplementary Table S5 available at *JXB* online). All the chloroplast biogenesis- and maintenance-related genes upregulated by low CO_2_ shown in Table 3 were also upregulated in C_4_ *Flaveria* species, and five genes downregulated by low CO_2_ (At5g52540, At1g52290, At5g20720, At2g32180, and At3g19820) were also downregulated in C_4_ *Flaveria* species (Gowik *et al.*, 2011); however, only At44446, At5g52540, At3g17040, and At1g52290 showed a ratio of expression abundance greater than 2.

**Table 3.** Transcript abundance of genes related to chloroplast biogenesis and maintenanceReads were mapped onto the latest *Arabidopsis thaliana* Col-0 genome assembly (gene mapping) or a minimal set of coding sequences of the TAIR 9 genome release (core set mapping) using bowtie. Low: low CO_2_, 100 ppm; Nor: normal CO_2_, 380 ppm; rpkm, reads per kilobase per million mapped reads.

Gene ID	Protein	Gene mapping			Core set mapping		
		Low (rpkm)	Nor (rpkm)	Fold change	Low (rpkm)	Nor (rpkm)	Fold change
At1g02560	CLPP5 (nuclear-encoded CLP protease 5), protease subunit	177.486	153.738	1.154	203.821	177.293	1.150
At1g06430	FTSH8 (cell-division protease ftsH-8)	46.648	33.676	1.385	53.561	38.836	1.379
At1g09340	CRB (chloroplast RNA binding)	477.334	377.221	1.265	548.065	435.050	1.260
At1g10350	Putative DnaJ heat-shock protein	5.668	8.818	0.643	6.507	10.169	0.640
At1g32080	Putative membrane protein	277.058	238.279	1.163	318.113	274.786	1.158
At1g44446	Chlorophyllide a oxygenase	16.090	45.126	0.357	18.475	52.075	0.355
At1g52290	Protein kinase-like protein	6.746	13.767	0.490	–	–	–
At1g55490	CPN60B (chaperonin 60 beta); RuBisCO large subunit-binding protein subunit beta	294.626	236.862	1.244	346.733	285.802	1.213
At1g62750	SCO1(SNOWY COTYLEDON1); elongation factor EF-G	369.741	244.662	1.511	443.343	295.894	1.498
At1g74730	Unknown protein	233.905	183.326	1.276	268.981	211.675	1.271
At2g03390	uvrB/uvrC motif-containing protein	43.955	31.538	1.394	50.468	36.370	1.388
At2g30950	VAR2 (VARIEGATED 2); cell-division protease ftsH-2	621.194	440.354	1.411	713.485	507.890	1.405
At2g32180	PTAC18 (plastid transcriptionally active 18)	19.442	27.606	0.704	22.323	31.836	0.701
At2g35490	Putative plastid-lipid- associated protein 3	108.463	88.003	1.232	124.626	101.487	1.228
At2g46100	Nuclear transport factor 2 (NTF2) family protein	63.534	49.599	1.281	72.948	57.419	1.270
At3g17040	HCF107 (high chlorophyll fluorescent 107)	9.314	21.102	0.441	10.694	24.335	0.439
At3g19820	DWF1 (DWARF 1)	77.847	87.660	0.888	89.382	101.091	0.884
At3g24430	HCF101 (high chlorophyll fluorescence 101)	65.051	49.942	1.303	74.690	57.594	1.297
At4g24190	SHD (SHEPHERD)/HEAT SHOCK PROTEIN 90–7	73.310	64.708	1.133	84.173	74.623	1.128
At5g12470	Unknown protein	52.805	35.339	1.494	60.629	40.753	1.488
At5g20720	CPN20 (chaperonin 20)	247.229	356.642	0.693	283.940	411.333	0.690
At5g42270	VAR1 (VARIEGATED 1); cell-division protease ftsH-5	413.651	316.477	1.307	475.001	364.966	1.301
At5g52540	Unknown protein	16.931	45.877	0.369	19.307	52.863	0.365

Of the genes showing a fold change more than 2, three (At1g44446, At3g17040, and At5g52540) were enriched in C_4_ *Flaveria* species compared to C_3_ species. PSII concentrations are well correlated with chlorophyll b synthesis (Bailey *et al.*, 2001), and chlorophyllide a oxygenase (At1g44446) is considered a critical enzyme responsible for chlorophyll b synthesis (Yamasato *et al.*, 2005). HCF107 (At3g17040) is a sequence-specific RNA-binding protein and remodels local RNA structure in a manner that accounts for its ability to enhance translation (Sane *et al.*, 2005; Hammani *et al.*, 2012). The *hcf107* mutation in *Arabidopsis* leads to a defective PSII (Felder *et al.*, 2001). Although many chloroplast-targeted DnaJ proteins have not been characterized, it has been hypothesized that chloroplast-targeted DnaJ proteins participate in protein folding, unfolding, and assembly processes, and some DnaJ proteins are involved in the stabilization of thylakoid membrane complexes such as photosystem II (Chen *et al.*, 2010). Therefore, these three downregulated genes were related to reduced PSII and this is in agreement with the ultrastructural analysis (Fig. 3).

### Differentially expressed transcription factors

Ten differentially expressed transcription factors were identified (|log_2_Ratio|≥1.2) (Table 4). Of these, *GOLDEN2-LIKE2* (*GLK2*, At5g44190), of the GLK family which is involved in chloroplast development (Langdale, 2011), was significantly downregulated under low CO_2_. The *GLK2* counterpart *GLK1* (At2g20570) was also downregulated in low CO_2_ but to a lesser extent.

**Table 4.** Differentially expressed transcription factors using Deseq softwareAP2-EREBP, Apetala 2 ethylene-responsive-element-binding proteins; C2H2, C2H2 zinc finger domain; G2-like, golden2-like; SBP, SQUAMOSA promoter-binding proteins. *P*<0.001, FDR<0.025, |log_2_Ratio|≥1.2.

TF family name	TF locus ID	Gene name	Gene description
Upregulated under low CO_2_			
AP2-EREBP	At1g74930	*ORA47* (*Octadecanoid derivative- responsive AP2/ERF-domain transcription factor 47*)	ORA47 is a regulator of jasmonate biosynthesis (Pauwels and Goossens, 2008)
C2C2-GATA	At4g26150	*CGA1* (*CYTOKININ-RESPONSIVE GATA FACTOR1*)	CGA1 was regulated by light, nitrogen, cytokinin, and gibberellic acid, and modulated nitrogen assimilation, chloroplast development, and starch production (Bi *et al.*, 2005; Naito *et al.*, 2007; Mara and Irish, 2008; Richter *et al.*, 2010; Hudson *et al.*, 2011); CGA1 play a key role in chloroplast development, growth, and divison in *Arabidopsis* (Chiang *et al.*, 2012)
AP2-EREBP	At4g34410	*RRTF1* (*redox-responsive transcription factor 1*)	RTF1 is involved in redox homeostasis under high light stress (Khandelwal *et al.*, 2008)
AP2-EREBP	At5g05410	*DREB2A (dehydration-responsive element-binding protein 2A)*	DREB2A is involved in dehydration- responsive gene expression and overexpression of an active form of DREB2A results in significant stress tolerance to dehydration and significant growth retardation (Sakuma *et al.*, 2006)
C2H2	At5g59820	*ZAT12*	Zat12 plays a central role in reactive oxygen and abiotic stress signalling in *Arabidopsis* and overexpression of Zat12 in *Arabidopsis* results in the enhanced expression of oxidative- and light stress-response transcripts (Davletova *et al.*, 2005)
Downregulated under low CO_2_			
C2C2-CO-like	At1g49130	*COL8* (CONSTANS-LIKE 8)	Zinc finger (B-box type) family protein
SBP	At2g33810	*SPL3* (*SQUAMOSA PROMOTER BINDING PROTEIN-LIKE 3*)	SPL3 is involved in regulation of flowering and vegetative phase change (Cardon *et al.*, 1997; Wu and Poethig, 2006; Yamaguchi *et al.*, 2009)
C2C2-CO-like	At4g27310	*BBX28*	Zinc finger (B-box type) family protein
G2-like	At5g44190	*GLK2* (*Golden2-like 2*)	GLK2 is required for normal chloroplast development (Fitter *et al.*, 2002); GLK2 together with GLK1 optimize photosynthetic capacity by integrating responses to variable enironmental and endogenous cues (Waters *et al.*, 2009)
MADS	At5g62165	*AGL42* (*AGAMOUS-LIKE 42*)	AGL42 is involved in the floral transition and RNAi-directed downregulation of AGL24 results in late flowering (Yu *et al.*, 2002)

Waters *et al.* (2009) identified 20 most upregulated genes by *GLK1* and *GLK2* induction using inducible gene expression combined with transcriptome analysis. The current work assessed the alteration of these 20 primary targets of *GLK* gene action and found nearly that all of them, except *COR15a* (At2g42540) were downregulated (Table 5 and Supplementary Table S6 available at *JXB* online). *COR15a* was significantly induced under low CO_2_ instead, possibly because COR15a is an indirect, secondary target of *GLK2* (Waters *et al.*, 2009).

**Table 5.** Transcript abundance of GLK-regulated genesReads were mapped onto the latest *Arabidopsis thaliana* Col-0 genome assembly (gene mapping) or a minimal set of coding sequences of the TAIR 9 genome release (core set mapping) using bowtie. The most upregulated genes by *GLK1* and *GLK2* induction identified by Waters *et al.* (2009) were examined and nearly all of them were downregulated by low CO_2_, except *COR15a* (At2g42540). Low: low CO_2_, 100 ppm; Nor: normal CO_2_, 380 ppm; rpkm, reads per kilobase per million mapped reads. CAO, chlorophyllide a oxygenase; CHLH, magnesium chelatase; COR15a, COLD-REGULATED 15A; GCN5 related, ornithine N-delta-acetyltransferase; GLK1, Golden2-like 1; GLK2, Golden2-like 2; Lhcb, light harvesting complex subunit; MRU1, mto responding up 1; PORB, NADPH:protochlorophyllide oxidoreductase B.

Gene ID	Protein	Gene mapping			Core set mapping		
		Low (rpkm)	Nor (rpkm)	Fold change	Low (rpkm)	Nor (rpkm)	Fold change
At1g15820	Lhcb6	2153.237	3099.576	0.695	2472.919	3575.038	0.692
At1g44446	CAO	16.090	45.126	0.357	18.475	52.075	0.355
At1g76100	Plastocyanin	132.858	261.423	0.508	–	–	–
At2g05070	Lhcb2.2	431.847	1425.637	0.303	–	–	–
At2g20570	GLK1	32.786	59.366	0.552	37.677	68.503	0.550
At2g34430	Lhcb1.4	599.406	1663.045	0.360	–	–	–
At2g35260	Expressed protein	74.776	98.821	0.757	85.888	114.002	0.753
At2g39030	GCN5 related	0.140	3.243	0.043	–	–	–
At2g42220	Rhodanese-like domain-containing protein	220.272	249.887	0.881	253.769	288.712	0.879
At2g42540	COR15a	193.204	31.607	6.113	268.502	47.764	5.621
At3g08940	Lhcb4.2	326.811	1274.137	0.256	–	–	–
At3g27690	Lhcb2.4	136.883	414.191	0.330	157.608	478.391	0.329
At3g56940	Mg-Proto IX ME cyclase	428.161	770.890	0.555	491.646	889.102	0.553
At4g27440	PORB	400.475	873.866	0.458	–	–	–
At5g13630	CHLH	456.682	432.350	1.056	524.401	498.633	1.052
At5g35490	MRU1	7.921	28.980	0.273	9.095	33.420	0.272
At5g44190	GLK2	5.722	26.922	0.213	6.570	31.047	0.212
At5g54270	Lhcb3	2240.644	3734.380	0.600	2573.689	4307.756	0.597

### Stress-induced mutagenesis pathway was changed under low CO_2_

It has been shown that DNA double-strand break-dependent stress-induced mutagenesis is important to evolution, through producing more mutations under stress in *Escherichia coli* (Cirz *et al.*, 2005; Shee *et al.*, 2011; Al Mamun *et al.*, 2012). As a severe stress, can low CO_2_ induce more mutagenesis in natural populations? This work examined the transcriptional changes in genes encoding products related to human DNA repair proteins and found that genes involved in damage sensing (At5g40450, At2g26980, At4g04720), photoreactivation (At3g15620), homologous recombination (At3g48190), nucleotide excision repair (At2g36490, At3g02060, At5g04560, At1g52500, At3g28030, At5g45400), and DNA polymerases (At4g32700, At1g67500) were upregulated by low CO_2_ (Table 6 and Supplementary Table S7 available at *JXB* online). These results suggest that low CO_2_ might induce a similar mechanism of DNA double-strand break-dependent stress-induced mutagenesis to promote evolution.

## Discussion

This study, as far as is known for the first time, investigated responses to low CO_2_ at the transcriptome level in model plant *Arabidopsis*. Here, the observed changes of transcriptomics under low CO_2_ are briefly discussed, with particular reference to their potential significance for fitness of C_3_ plants under low CO_2_ and potential linkage to C_4_ photosynthesis evolution.

### Low CO_2_ reduced productivity

*Arabidopsis* plants grown under low CO_2_ had extremely small stature compared with plants grown under normal CO_2_ (Fig. 1). This result is in accordance with previous studies on the effect of low CO_2_ on plant growth (Ward, 2005). *Arabidopsis* grown under low CO_2_ has about a 7-day delay in flowering time. This has also been observed earlier (Ward and Strain, 1997) and could be interpreted as a mechanism to allow for greater accumulation of stored reserves that could be allocated to reproduction, resulting in increased fitness under low CO_2_ (Sage and Coleman, 2001; Ward, 2005).

Many studies have shown that atmospheric CO_2_ concentration negatively regulates stomatal density (Woodward, 1987; Beerling *et al.*, 2001; Franks and Beerling, 2009; Doheny-Adams *et al.*, 2012; Franks *et al.*, 2012). Paleontological research has suggested that the long-term decreases in atmospheric throughout the entire evolutionary history of vascular plants led to the evolution of high densities of small stomata in order to attain the highest *g*_cmax_ values required to counter CO_2_ ‘starvation’ (Franks and Beerling, 2009; Franks *et al.*, 2012). Stomata also exhibit short-term adaptive responses to atmospheric CO_2_ over much shorter timescales. For example, *A. thaliana* Col-0 grown at high CO_2_ (720 ppm) had reduced stomata density compared with those grown at ambient CO_2_ (360 ppm) (Lake *et al.*, 2001). In the current work, plants grown under low CO_2_ developed leaves with higher stomatal density (over 60% increase compared to normal CO_2_; Fig. 2), suggesting that the plants developed a greater *g*_cmax_ to counteract the CO_2_ limitation of photosynthesis. These results suggest that low CO_2_ is a severe stress to C_3_ plants and may greatly reduce C_3_ plant productivity.

### Responses of genes involved in C_4_ photosynthesis and photorespiration under low CO_2_

In C_4_ plants, CO_2_ is initially fixed by the enzyme PEPC into a C_4_ acid and then transported to the site of Rubisco (Hatch, 1987). The only photosynthetic gene expression patterns common to all independently evolved C_4_ lineages are upregulation of PEPC and downregulation of Rubisco in mesophyll cells (Sinha and Kellogg, 1996; Langdale, 2011). *Arabidopsis* has four genes encoding PEPC, and AtPPC2 (At2g42600) is the only isoform expressed in leaves. Unlike the other three PEPCs, the expression of AtPPC2 is stable and has not been reported to be regulated by any stress (Sánchez *et al.*, 2006; Doubnerová and Ryšlavá, 2011); however, the current work found that AtPPC2 was upregulated by low CO_2_ (Table 1). The regulators of photosynthetic genes are also crucial to maintain C_4_ photosynthesis: e.g. plant PEPC activity is further regulated through reversible phosphorylation by PEPC-K (Nimmo, 2003). Transcripts encoding the C_4_-specific regulatory factors PEPC-K and pyruvate orthophosphate dikinase regulatory protein were upregulated as well (Table 1). However, changes in other C_4_-related genes were less, with fold changes of less than 2.

When grown in low CO_2_, plants would experience relatively high levels of flux through the photorespiratory pathway because of the competitive reactions of Rubisco oxygenation. In this study, a trend of upregulation of the photorespiratory genes was observed in plants grown under low CO_2_ (Table 2), although most of the genes showed a fold change of less than 2. The recent study of transcriptome analysis using C_3_, C_3_–C_4_ intermediate, and C_4_ species of *Flaveria* found that transcript abundances for most genes related to photorespiration in the C_3_–C_4_ intermediate species *Flaveria ramosissima* were even higher than in the C_3_ species *Flaveria robusta* (Gowik *et al.*, 2011), which is indicative of the importance of the photorespiratory pathway during the evolution of C_4_ photosynthesis. The different subunits of glycine decarboxylase showed altered expression, although the fold changes of these subunits were about 0.9–1.3.

Overall, the data from this study suggest that expression of PEPC and PEPC-K is increased under low CO_2_, which most likely reflects their potential role for refixation of photorespired CO_2_ under low CO_2_ (Sage *et al.*, 2012). For most of the other C_4_ genes, although trends of upregulation were observed, the fold changes were less than 2. Although by using expression level changes of all genes under two conditions as background, this work obtained *P*-values much less than 0.01 for many C_4_-related genes, it is likely that lack of biological replicates could had potentially led to an overestimation of the reliability of statistical tests and caused problems in identifying significantly changed genes, especially when their fold changes were less than 2. Based on these, this work cannot state that low CO_2_ induced upregulation of C_4_ genes, except for those genes which showed fold changes over 2 (e.g. PEPC).

### Readjustment of balance between light absorption and CO_2_ fixation under low CO_2_

These data on chloroplast ultrastructure and transcript abundance of genes involved in chloroplast biogenesis and maintenance are consistent with the model for long-term photosynthetic regulation by GLK proteins (Waters and Langdale, 2009). When light is high and atmospheric CO_2_ is limiting, the rate of CO_2_ fixation is insufficient to use all of the output of the light-harvesting reactions, resulting in an overly reduced photosynthetic electron transport. This triggers a decrease of GLK transcription (GLK1 and GLK2; Table 5). Since GLK transcription factors directly regulate a large suite of genes involved in light-harvesting and thylakoid protein complexes, especially those of PSII (Waters *et al.*, 2009), the light-harvesting components in the thylakoid membrane LHCB2.2 (At2g05070), LHCB4.2 (At3g08940), Lhcb3 (At5g54270), Lhcb2.4 (At3g27690), and Lhcb1.4 (At2g34430) were downregulated under low CO_2_. In addition, the downregulation of the chlorophyllide a oxygenase gene led to the decrease of chlorophyll b synthesis. These results were consistent with the fewer and less-stacked grana observed and a higher proportion of non-stacked stromal lamellae, as observed in *glk1 glk2* mutants (Fig. 3). Therefore, these observed expression changes in GLK and the genes regulated by GLK can be interpreted as reflecting the altered balance between CO_2_ fixation and light absorption.

### Evolutionary implications of plants of to low CO_2_

Growing evidence suggests that all of the basic elements of C_4_ photosynthesis already existed in C_3_ plants. For example, all of the enzymes involved in C_4_ photosynthesis exist in C_3_ plants and play different roles in C_3_ plant metabolism (Aubry *et al.*, 2011). Some elements controlling the cell specific expression of C_4_-related enzymes have been found in C_3_ plants (Brown *et al.*, 2011). Moreover, typical C_3_ plants (e.g. tobacco and *Arabidopsis*) show the characteristics of C_4_ photosynthesis in midveins (Hibberd and Quick, 2002; Brown *et al.*, 2010).

Can some features related to C_4_ photosynthesis be enhanced under some conditions in a C_3_ plant? This work showed that under low atmospheric CO_2_, *A. thaliana* Col-0 adjusted a series of biological processes, especially the upregulation of PEPC and PEPC-K gene expression, and also the altered expression of some transcription factors related to photosynthesis development, and the downregulation of light-harvesting and thylakoid protein complexes. Although this study also observed that the majority of the other C_4_-cycle genes were upregulated under low CO_2_ in *Arabidopsis*, their fold changes were less than 2 and therefore no firm statements regarding their changes can be made.

Experiments with more biological replicates and *Arabidopsis* accessions are still needed to firmly conclude whether low CO_2_ can induce upregulation of other C_4_-related genes. Therefore, the results from this paper do not support a scenario where low CO_2_ acts as a signal to induce C_4_ biochemical features in C_3_ plants. It is most likely that the upregulation of PEPC and PEPC-K might be a mechanism that C_3_ plants used to refix photorespired and respired CO_2_ and also to recapture the released ammonium from photorespiration and hence increase the competitive advantages under low CO_2_ conditions.

## Supplementary material

Supplementary data are available at *JXB* online.

Supplementary Fig. S1. Measurement of stomatal density.

Supplementary Table S1. Gene mapping results.

Supplementary Table S2. Core-set gene mapping results.

Supplementary Table S3. Transcript abundance of C_4_ cycle genes and C_4_-related transporters.

Supplementary Table S4. Transcript abundance of photorespiration genes.

Supplementary Table S5. Transcript abundance of genes related to chloroplast biogenesis and maintenance.

Supplementary Table S6. The 20 most-upregulated genes following GLK2 induction.

Supplementary Table S7. Transcript abundance of DNA-repair genes.

Supplementary Table S8. Transcript abundance of photosynthesis genes.

Supplementary Table S9. Transcript abundance of Calvin Benson cycle genes.

Supplementary Table S10. Transcript abundance of ABA-metabolism genes.

Supplementary Table S11. Transcript abundance of auxin-metabolism genes.

Supplementary Data
