# Supplementary material for: Was low CO2 a driving force of C4 evolution: Arabidopsis responses to long-term low CO2 stress
Source: J Exp Bot. 2014 May 22;65(13):3657–67. doi: 10.1093/jxb/eru193 (PMC4085967; doi:10.1093/jxb/eru193)
Supplement: Supplementary Data [file supp_65_13_3657__index.html]

Was low CO2 a driving force of C4 evolution: Arabidopsis responses to long-term low CO2 stress — Supplementary Data 

# Was low CO2 a driving force of C4 evolution: *Arabidopsis* responses to long-term low CO2 stress

## Supplementary Data

Data files

**Files in this Data Supplement:**

- Supplementary Data - Supplementary Data
- Supplementary Data - Supplementary Data
