# Supplementary material for: Was low CO2 a driving force of C4 evolution: Arabidopsis responses to long-term low CO2 stress
Source: J Exp Bot. 2014 May 22;65(13):3657–67. doi: 10.1093/jxb/eru193 (PMC4085967; doi:10.1093/jxb/eru193)

Was low [CO2] a driving force of C4 evolution? *Arabidopsis* responses to long-term low [CO2] stress. *Yuanyuan Li, Jiajia Xu, Hui Zhang, and Xin-Guang Zhu*

Supplement Figure S1. Measure stomata density

We examined eight fully expanded leaves from eight individual plants for each treatment. For each leaf, we took photos from 4 fields (same size; position as indicated in the following figure, red rectangle) of abaxial (lower) leaf blade epidermis. So for each treatment (normal [CO_2] and low [CO_2]), we measured 32 area (n=32).

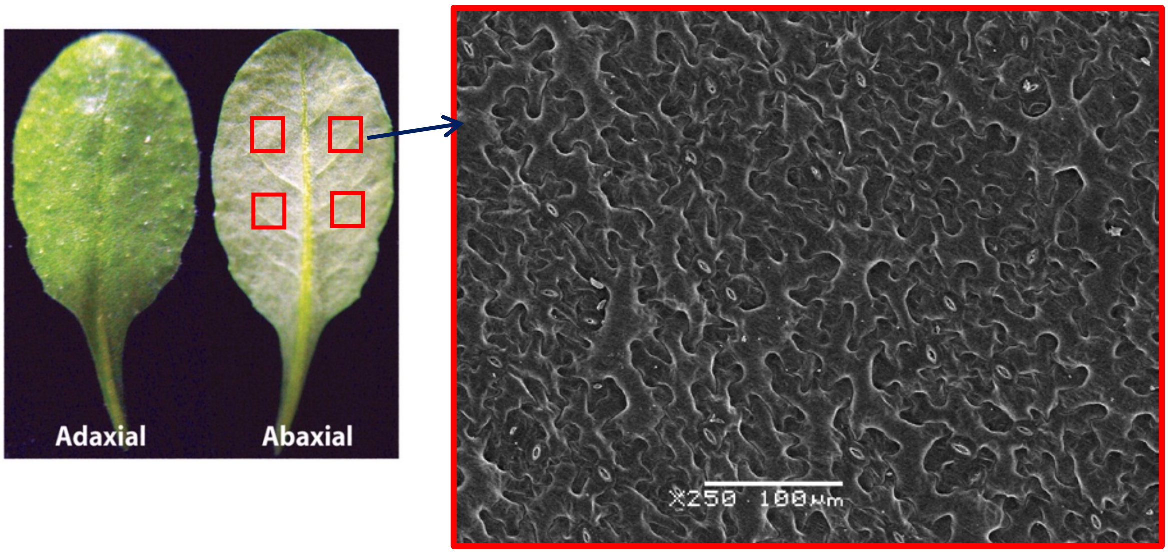

Supplement: Supplementary Data [file supp_eru193_jexbot115923_file002.pdf]
